# The regulatory function of *Blastocystis* spp. on the immune inflammatory response in the gut microbiome

**DOI:** 10.3389/fcimb.2022.967724

**Published:** 2022-08-31

**Authors:** Liliana Rojas-Velázquez, Patricia Morán, Angélica Serrano-Vázquez, Tobías Portillo-Bobadilla, Enrique González, Horacio Pérez-Juárez, Eric Hernández, Oswaldo Partida-Rodríguez, Miriam Nieves-Ramírez, Angeles Padilla, Martha Zaragoza, Cecilia Ximénez

**Affiliations:** ^1^ Unidad de Investigación en Medicina Experimental, Facultad de Medicina, Universidad Nacional Autónoma de México (UNAM), Mexico City, Mexico; ^2^ Red de Apoyo a la Investigación, Coordinación de la Investigación Científica, Universidad Nacional Autónoma de México (UNAM) e Instituto Nacional de Ciencias Médicas y Nutrición Salvador Zubirán, Mexico City, Mexico

**Keywords:** *Blastocystis*, immune inflammatory response, regulatory function, gut microbiome, human gut microbiota

## Abstract

*Blastocystis* spp. is a unicellular organism that resides in digestive tract of various vertebrates, with a worldwide distribution and a variable prevalence. For many years, *Blastocystis* spp. was considered a cyst of a flagellate, a fungus, or a saprophyte yeast of the digestive tract; in 1996, it is placed in the group of stramenopiles (heterokonts). Since its new classification, many questions have arisen around this protist about its role as a pathogen or non-pathogen organism. Recent evidence indicates that *Blastocystis* spp. participates in the immune inflammatory response in the intestinal microbiome generating an anti-inflammatory response, showing a lower concentration of fecal inflammatory markers in infected human hosts. Here, we review recent findings on the regulatory function of *Blastocystis* spp. in the immune inflammatory response to comprehend the purpose of *Blastocystis* spp. in health and disease, defining if *Blastocystis* spp. is really a pathogen, a commensal or even a mutualist in the human gut microbiome.

## Introduction

*Blastocystis* spp. (Heterokonta or Stramenopiles) is an enteric protist. It has a worldwide distribution that inhabiting the digestive tract of several vertebrates, being the most prevalent protist of the human intestine (Silberman et al., 1996; Stenzel and Boreham, 1996; Tan, 2008). *Blastocystis* spp. (named *Blastocystis* hereafter) exhibits great genetic diversity; by the moment, 26 subtypes have been established on the basis of the small subunit of the ribosomal RNA gene (SSU rRNA), and each subtype has a distinct distribution and different types of host around the world (Aynur et al., 2019; Maloney et al., 2019). So far, it has been reported that humans could be infected by specific subtypes as ST1 to ST10 and ST12, but an undoubted fact is that ST1, ST2, ST3, and ST4 are the most frequently subtypes identified in humans (Moosavi et al., 2012; Alfellani et al., 2013; Fontanelli Sulekova et al., 2019; Jiménez et al., 2019). The pathogenicity or non-pathogenicity of *Blastocystis* depends on several factors such as the interaction with the intestinal microbiota, the infecting subtype, and the host’s immune response. Some studies carried out in different settings suggest that *Blastocystis* is part of the normal gut microbiota of humans and other mammals, being able to colonize the intestinal tract and establish itself for prolonged periods without causing disease (Parfrey et al., 2011; Scanlan et al., 2014; Pandey et al., 2015). An interplay with the immune system is established. There is evidence, for example, that *Blastocystis* colonization may be related to an anti-inflammatory response favoring changes in the bacterial composition of the gut microbiota, increasing levels of Firmicutes and promoting a greater bacterial diversity (Nourrisson et al., 2021). Our group reported that asymptomatic healthy individuals infected with *Blastocystis* show an anti-inflammatory response characterized by lower concentration of fecal inflammatory markers and a higher alpha diversity in the bacterial community (Partida-Rodríguez et al., 2017; Nieves-Ramírez et al., 2018).

On the other side is the issue of *Blastocystis* pathogenicity or non-pathogenicity related to genetic differences of the subtypes (Stensvold et al., 2009; Wu et al., 2014). Some subtypes had been associated with unhealthy changes in humans as the case of ST1, ST4, and ST7 and, on the contrary. ST3 has been related as a non-pathogenic subtype (Domínguez-Márquez et al., 2009; Eroglu et al., 2009; Stensvold et al., 2009). However, it is early to say which subtypes are pathogenic or not based on the little information that is currently available. In addition, it was demonstrated that some subtypes could alter the gut microbiome, for example, ST7 produces a decrease in beneficial bacterial populations, such as *Bifidobacterium* and *Lactobacillus* (Yason et al., 2019), and ST3 was associated to an eubiotic state characterized by beneficial species that are members of the phyla Firmicutes and Bacteroidetes, such as those of the genera *Ruminococcus* and *Prevotella*, respectively (Iebba et al., 2016a).

Some initial reports indicate that *Blastocystis* infection might be related to the inflammatory state of the intestine that is typical of the irritable bowel syndrome (IBS) (Giacometti et al., 1999). As far as one knows, the associated symptoms of *Blastocystis* infection are the outcome of the innate immune response that follows the breakdown of the intestinal barrier. There is infiltration and damage of the intestinal epithelium that involves activation of membrane receptors such as TLRs and CD8 T lymphocytes, macrophages, and neutrophils activation, including Immunoglobulin M (IgM), IgG, and IgA production (Vitetta et al., 2016). However, the function of *Blastocystis* colonization associated with gastrointestinal symptoms remains unresolved.

Apparently, *Blastocystis* has developed ways to take advantage of the host immune inflammatory response to settler and to continue host colonization without causing disease. Here, we review recently described strategies by which *Blastocystis* could regulate the immune inflammatory response in the gut microbiome.

## The effect of *Blastocystis* on the immune inflammatory response

The human gut microbial community comprises a highly complex ecosystem (The Human Microbiome Project Consortium, 2012). Bacteria, nematodes, and protozoan parasites are common in the gastrointestinal tract. They have co-evolved and adapted to a variety of circumstances in an interplay with the host, so it is not surprising that they become important elements as regulators and/or modulators of the host immune response (Reynolds et al., 2015). Under certain conditions, this can undermine the host ability to initiate an effective immune safeguarding mechanism, allowing the colonization and persistence of infection of parasites and other microorganisms (Round and Mazmanian, 2010; Bancroft et al., 2012; Glendinning et al., 2014), which adapt to the new ecological niches, as could be the scenario of *Blastocystis*.

In the intestine, *Blastocystis* has an interplay with the intestinal epithelium and the underlying immune system (Belkaid and Hand, 2014). IgA is the most abundant mucosal antibody that has a fundamental function conserving homeostasis with the microbiome by joining and neutralizing invading pathogens near the mucus layer (Gutzeit et al., 2014). IgA secretion in the intestinal lumen is caused by parasitic infections with helminths to limit the fertility of the parasite and provide immune protection against reinfections (Johansen et al., 1999; McCoy et al., 2008). Individuals colonized with *Blastocystis* have presented lower levels of fecal IgA compared with non-colonized individuals (Nieves-Ramírez et al., 2018). *Blastocystis* is also correlated with decreased neutrophil counts in blood (Cheng et al., 2003) and is known to produce serine proteases that degrade secretory IgA (sIgA) (Puthia et al., 2005). In addition, individuals colonized by *Blastocystis* displayed lower levels of fecal calprotectin (Nieves-Ramírez et al., 2018). The calprotectin is a protein used as a marker of intestinal inflammation and is derived from the secretion of cytosolic proteins from neutrophils (Walsham and Sherwood, 2016). It seems that the interaction of *Blastocystis* in the intestine stablished an anti-inflammatory habitat.

Serum cytokines have been reported in cultures of *in vitro* cell lines incubated with *Blastocystis* that favor the production of interleukin-8 (IL-8) and granulocyte-macrophage colony-stimulating factor (Long et al., 2001). In a murine model through histopathological examinations, the pathogenicity of *Blastocystis* and its capacity to modulate the immune response were evaluated, finding changes in the epithelia, with an exfoliation and inflammatory cell infiltration in the submucosa, severe hyperplasia of caliciform cells, and *Blastocystis* infiltrated in all layers of the large intestine. In addition, mice infected with *Blastocystis* show a greater expression of cytokine IL-12 and tumor necrosis factor–alpha (TNF-α) and a lower expression of cytokine IL-4 and IL-10 (Abdel-Hafeez et al., 2016). Meanwhile, it was demonstrated that *Blastocystis* ST7 induces the expression of IL-1β and IL-6 proinflammatory cytokines through the activation of the mitogen-activated protein kinases in mouse intestinal explants (Lim et al., 2014). In addition, in an experimental model that used mice colonized with ST4, the production of short-chain fatty acids (SCFAs) and the proportion of anti-inflammatory cytokine IL-10 were increased (Deng and Tan, 2022). Meanwhile, in patients with IBS infected with *Blastocystis* subtypes ST1, ST2, and ST3 through the evaluation of serum by enzyme-linked immunosorbent assays, it was found that there was an increase in the concentration of the cytokines IL-6 and TNF-α (Azizian et al., 2016).

On the basis of these studies and although they are scarce until now, it seems that *Blastocystis* and some specific subtypes could generate an anti-inflammatory scenario, of course, future research on this issue is required to elucidate how this microorganism interacts with the host immune inflammatory response.

## The modulation of *Blastocystis* on the intestinal microbiota and its interaction with the immune inflammatory response

The microorganisms in healthy intestinal microbiota consist of trillions of virus, bacteria, and other protozoa and fungi that are all in proximity with the intestinal mucosa, developing specific and important biological interactions and physiological functions critical for the host (Sekirov et al., 2010; Brown et al., 2013). This long-term and dynamic coevolution with the human intestinal immune system eventually permits or favors mutualistic host-microbial relationships that have significant impact to the maturation, development, and modulation of the immune response. There is a fine regulation of the expression of immune mediators that impact the recruitment and differentiation of local immune cell populations in harmony with microorganisms and that direct the establishment of the intestinal microbiome (Sjögren et al., 2009; Caballero and Pamer, 2015).

Likewise, intestinal parasites such as helminths and protozoa regularly secrete molecules that modify the ecological niche and therefore can modulate the structure and function of the gut microbiota (Sekirov et al., 2010). The intestinal environment can be interpreted from different levels of interactions between biotic and abiotic factors that have an impact on biochemical networks, microbial communities, and the function of the host immune system. An example is the human plasminogen’s increased effect on *Bifidobacterium* cell adhesion to enterocytes or bacteria role as modulator of cell behavior in immune and inflammatory processes (Candela et al., 2008; Keragala and Medcalf, 2021). Only recently, the relationship between human-associated gut protists and the gut bacterial community has been explored to elucidate their role in dysbiosis or pathogenesis (Burgess and Petri, 2016; Barash et al., 2017). We are in the process of understanding the key factors that modify the balance between health and disease in relation to the diversity of the microbiome and its changes in the composition, structure, and function (Brown et al., 2013). One of the evident difficulties is that the intestinal microbiota behavior can be highly variable in the human host, determined by various factors such as diet, sociodemographic status, health and disease condition, or the use of antibiotics and, to a lesser extent, by the genetic component (Cho and Blaser, 2012; Yatsunenko et al., 2012; Goodrich et al., 2016).

Currently, *Blastocystis* is clearly associated with changes in the composition of the microbiota in the human host (**Table 1**). The unavoidable question is whether these protozoa are capable of regulating the intestinal inflammatory immune response in humans modulating bacterial populations, if so, by which mechanisms they act to do it. This will remain controversial until new research emerged. So far, it has been found that *Blastocystis* colonization is strongly related with broad shifts in the gut-resident bacterial community and with an increase in bacterial diversity (Nieves-Ramírez et al., 2018; Deng et al., 2021). Bacterial diversity is associated to healthy microbiota and favorable immune response in the host (The Human Microbiome Project Consortium, 2012); some hypotheses state that *Blastocystis* could have a predatory effect on bacteria population, feeding on abundant taxa and modifying in this way the diversity, increasing or decreasing bacterial populations (Matz and Kjelleberg, 2005; Kurm et al., 2019). In a previous study, we observed that *Blastocystis* was associated with an increment of *Ruminococcaceae bromii* (Nieves-Ramírez et al., 2018), which is well known for its ability to degrade resistant starches in the human gut (Ze et al., 2012). Data suggested that microbial fermentation of resistant starches in the colon leads to SCFAs, such as lactic acid, acetate, propionate, and butyrate, which are the major end products of the microbial fermentation pathway (Ze et al., 2012; Maier et al., 2017; Pushpanathan et al., 2019; Tan et al., 2021; Sobh et al., 2022). Butyric acid is an important resource for nourishing the colonocytes and maintains healthy the colonic epithelium. Propionic acid and acetic acid have a protective effect lowering the pH in the large intestine, preventing the growth of pathogenic microorganisms and promoting the proliferation of beneficial bacteria (Shen et al., 2017). An adequate degradation of resistant sugars prevents chronic and inflammatory diseases [e.g., IBS, irritable bowel disease (IBD), and ulcerative colitis (UC)] (Ott, 2004; Nishida et al., 2018; Pushpanathan et al., 2019), so it possible that *Blastocystis* can indirectly regulate proinflammatory and inflammatory cytokines by modulating the intestinal microbiota.

**Table 1:** Modifications of *Blastocystis* spp. on the gut microbiota.

*Blastocystis* subtype	Country	Origin of samples	Clinical state	Bacterial		Gut microbiota composition		Reference
				Richness	Diversity	Increase	Decrease	
ST1 to ST4	France	Humans	IBS and no GI (ASI)	–	–		*Faecalibacterium prausnitzii*, *Bifidobacterium* sp.	(Nourrisson et al., 2014)
ST1 to ST4 and ST6	Spain and Denmark	Humans	IBS and ASI	↑	–	*Prevotella* and *Ruminococcus* enterotypes		(Andersen et al., 2015)
NS-ST	Denmark	Humans	ASI	–	–	*Prevotella*	*Bacteroides* and clostridial cluster XIVa	(O’Brien Andersen et al., 2016)
*Blastocystis*	France	Humans	IBS, IBD, GI and ASI	–	↑	Clostridia and Mollicutes (classes), Lactobacillales, Clostridiales (orders), Ruminococcaceae, and Prevotellaceae (families)	Enterococcaceae, Streptococcaceae, Lactobacillaceae, and Enterobacteriaceae (families)	Audebert et al., 2016
ST1 to ST8	Australia	Humans	IBS, IBD and ASI	–	–	Non-significant	Non-significant	(Nagel et al., 2016)
*Blastocystis*	Côte d´lvoire	Humans	ASI and GI	–	–	*F*. *prausnitzii*/*Escherichia coli* ratio		(Iebba et al., 2016)
*Blastocystis*	VC	Humans	Colorectal cancer, type 2 diabetes, liver cirrhosis obesity and IBD	–	–	Clostridiales, Firmicutes, and archaea organisms (*Methanobrevibacter smithii*)	*Bacteroides*and Proteobateria	(Beghini et al., 2017)
ST1 to ST4 and ST8	Sweden	Humans	ASI	↑	–	*Sporolactobacillus* and *Candidatus carsonella*	*Bacteroides*	(Forsell et al., 2017)
ST2 and ST3	Mexico	Humans	GI andASI	↑	–	*Prevotella copri*, *Ruminoccoccus bromii*, *Debaryomyces hansenii*, *Mucor mucedo*, *Aspergillus flavus*, *Mucor racemosus*, and *Issatchenkia terricola*	*Hymenolepis nana*	(Nieves-Ramírez et al., 2018)
ST1 to ST4 and ST7, ST8	Belgium	Humans	IBD and ASI	↑	↑		*Bacteroides* enterotype, *Akkermansia*	(Tito et al., 2019)
ST7	Singapore	Mouse model (human donor)	–	–	–		*Lactobacillus* and *Bifidobacterium*	(Yason et al., 2019)
*Blastocystis*	Mali	Humans	ASI	↑	↑	Firmicutes, Elusimicrobia, Lentisphaerae, and Euryarchaeota (phylum); *F*. *prausnitzii* and *Roseburia* sp.	Actinobacteria, Proteobacteria, unassigned bacteria, and Deinococcus–Thermus	(Kodio et al., 2019)
*Blastocystis*	India	Humans	VL	–	↑	Clostridiales vadin BB60	*Bacteroidaceae* and *Escherichia–Shigella*	(Lappan et al., 2019)
ST1 to ST4 and ST7	Italy	Humans	IBD, IBS, and chronic diarrhea	–	↑	*Prevotella*, *Methanobrevibacter* and *Ruminococcus*	*Bacteroides*	(Gabrielli et al., 2020)
*Blastocystis*	Colombia	Humans	ASI	↑	–	*Prevotella*	*Akkermansia*	(Alzate et al., 2020)
*Blastocystis*	Colombia	Humans	ASI	↑	↑	*Faecalibacterium*	*Prevotella*, *Bacteroides*, and *Akkermansia*	(Castañeda et al., 2020)
ST3		rat model (human donor)	Colitis	–	–	Clostridiales (Firmicutes),*Bilophila*, and *Butyricimonas*	Bacteroidales (*Bacteroidetes)*,Defluviitaleaceae	(Billy et al., 2021)
ST1 to ST3 and ST6	VC2	Humans	AS and type 1 diabetes	↑	–	Ruminococcaceae	*Bifidobacterium*	(Cinek et al., 2021)
*Blastocystis*	Côte d´lvoire	Humans	ASI	↑	↑	*Succinivibrio*	Bacteroides-driven enterotype	(Di Cristanziano et al., 2021)
ST1 to ST4	Cameroon	Humans	ASI	↑	–	Clostridiales	*Bacteroides-*driven enterotype	(Even et al., 2021)
*Blastocystis*	France	Humans	IBS and ASI	↓	–	Fumicutes, Bacteroidetes, Ruminococcaceae, Tenericutes, and Dipodascaceae	Aspergillaceae, *Aspergillus*, and *Penicillium*	(Nourrisson et al., 2021)
*Blastocystis*	Korea	Humans	ASI	↑	–	Clostridia, Ruminococcaceae, Prevotellaceae, and Faecalibacterium		(Kim et al., 2021)
ST1 to ST5 and ST7	Mexico	Humans	ASI and metabolic ill	–	–		Firmicutes/BacteroidetesRatio	(Muñoz Yañez et al., 2021)
ST2 to ST3 and ST5	Colombia	Humans	*C*. *difficile* infected, ASI, and diarrhea	↓	–	Saccharomycetales family Ascomycota and Basidiomycota Prevotellaceae*Faecalibacterium*, *Dorea*, and Lachnospiraceae	*Akkermansia*	(Vega et al., 2021)
*Blastocystis*	VC3	Humans	ASI	↑	–	Firmicutes, Bacteroidetes, *Prevotella*, *Faecalibacterium*, *Flavonifracter*, *Clostridium*, *Succinivibrio*, and *Oscillibacter*		(Stensvold et al., 2022)
ST4	Singapore	Wistar rats (*in vitro*)	ASI	–	–		*Bacteroides vulgatus*	(Deng and Tan, 2022)

VC, various countries; Italy, Tanzania, USA, Denmark, China, Mongolia, Germany, Spain, Peru, and France.

VC2, Azerbaijan, Jordan, Nigeria, Sudan, Tanzania, and Czechia.

VC3, Algerian, Egypt, Turkey, United Kingdom, and Denmark.

IBS, irritable bowel syndrome; IBD, irritable bowel disease.

GI, diarrhea, vomiting, bloating, constipation, and abdominal pain.

ASI, asymptomatic infected.

VL, visceral Leishmaniasis.

Other way on which *Blastocystis* could affect the abundance and diversity of the bacterial microbiota is interacting with the intestinal epithelium and the underlying immune tissue express cysteine proteases that cleave sIgA and secrete anti-lysozyme and anti-lactoferrin-factors that lead to immune evasion (Puthia et al., 2005; Gutzeit et al., 2014). Experiments with mice in cohousing and fecal transfer have been noted differences in IgA production, where the species with high content of IgA acquired the microbiota of species with low level of IgA suffering from a decrease in the levels of this immunoglobulin, which corroborates the regulatory capacity of IgA in the bacterial microbiome (Moon et al., 2015).

Recently, the existence of studies regarding to specific *Blastocystis* subtypes opens a new pathway to understand a little more how this microorganism exert a modulation on the microbiota intestinal population and its repercussion in the immune inflammatory response, for example, ST3 creates an eubiotic state characterized by favorable species of the phyla Firmicutes and Bacteroidetes (Andersen et al., 2015; Iebba et al., 2016a; Nieves-Ramírez et al., 2018; Gabrielli et al., 2020). The deterioration of the integrity of the microbiota and its barrier function favors the invasion of opportunistic and exogenous pathogens, generating dysbiosis, stimulating an inflammatory reaction of the host, and leading to a disorder of the intestinal nutritional environment and, frequently, to secretory diarrhea, with serious effects on the microbiota ecosystem. Therefore, it is of interest to preserve a eubiotic condition in the intestinal microbial ecosystem to guarantee a good state of health (Iebba et al., 2016a). Hence, again, ST3 indirectly could help in the immune response of the host, promoting the increased of beneficial bacterial populations.

In the case of ST7 that seems behave as a pathobiont, it produces a decrease in *Bifidobacterium* and *Lactobacillus* populations, which are considered as beneficial bacteria (Yason et al., 2019). ST7 was shown to have significantly greater cysteine protease activity compared with ST4 (Wu et al., 2014). Blastocystis ST7 has been shown to be more resistant to anti-parasitic drugs (Mirza et al., 2011; Yason et al., 2018) and against the host innate immune response (Yason et al., 2016). Some functions of *Bifidobacterium* are to maintain the epithelial barrier and to exert anti-inflammatory properties that can reduce the production of pro-inflammatory cytokines like IL-6 and TNF-α (Ling et al., 2016). In contrast, ST7 disrupts epithelial barrier and increases the levels of pro-inflammatory cytokines to trigger an inflammatory response (Long et al., 2001; Lim et al., 2014). *Lactobacillus* has also been found to significantly increase IgA levels (Carasi et al., 2015). Epidemiological studies have shown that reductions in *Lactobacillus* and *Bifidobacterium* contribute to increased susceptibility to gastrointestinal disorders, for example, patients with UC and Crohn´s disease CD had lower levels of *Lactobacillus* and *Bifidobacterium* populations, respectively (Jonkers, 2003; Ott et al., 2008). On this basis, *in vivo* studies have been carried out using the dextran sulfate sodium (DSS) colitis mouse model, in which an improvement in both colitis symptomatology and mucus production was observed after the administration of *Lactobacillus* and *Bifidobacterium* (Abdelouhab et al., 2012; Toumi et al., 2013). Therefore, a reduction of both intestinal bacteria would eliminate a protective element of the intestinal epithelium, which would provide an ideal environment for the pathogenesis of *Blastocystis*.

In contrast, ST4 *in vitro* plays a similar probiotic role, inhibiting the capacity of *Bacteroides valgatus* to compromise the intestinal epithelial barrier (Deng and Tan, 2022). *Bacteroides vulgatus* can produce mucin-degrading enzymes such as glycosidase, sialidases, and neuraminidase, which can profoundly weaken the mucosal barrier function and exaggerate inflammation (Ohkusa et al., 2009; Derrien et al., 2010). *In vitro* experiments demonstrated that *B. vulgatus* can invade colonic epithelial cells (SW-480 and HT-29) and activate the expression of pro-inflammatory cytokines (Ohkusa et al., 2009). Interestingly, Deng et al. reported in a second work (Deng et al., 2022) that ST4-altered microbiota from *Rag1* ^−/−^ mice reduces inflammation in experiment-induced colitis through an increase in “beneficial” microbes such as *Akkermansia*. Bacteria belonging to *Akkermansia* are associated with gut health, and the expansion of *Akkermansia* can increase mucus production to ameliorate intestinal inflammation (Everard et al., 2013). Moreover, fecal microbiota transplantation (FMT) from ST4-colonized mice increased the SCFA production and the proportion of anti-inflammatory cytokine IL-10 more profoundly than FMT from control mice. *Blastocystis* ST4 improves the intestinal inflammation in a mouse model. They also observed that *Blastocystis* ST4 colonization activates Th2 immune responses in normal healthy mice and DSS-induced colitis mice. It has been determined that Th2 cells are important sources of type 2 cytokines (IL-4, IL-5, and IL-13) and are also important effector cells during the inflammatory process (Gause et al., 2020). In addition, they also found that *Blastocystis* ST4 colonization increases the number of IL-10–producing Treg in the colonic mucosa of DSS-induced mice. The cytokine IL-10 produced by Treg cells is required for containment of inflammatory responses in mucosal tissues (Rubtsov et al., 2008). Both humans and mice deficient in IL-10 or IL-10 receptor are prone to develop severe intestinal inflammation (Glocker et al., 2009; Begue et al., 2011). Furthermore, we can agree more with them that future studies should focus on understanding the mechanistic connection between *Blastocystis* ST4 colonization, IL-10 signaling, and bacterial-derived SCFAs using relevant animal models, not just for this subtype, otherwise for all subtypes of *Blastocystis* that infect the human population.

Although there is still little information about the modulatory function of *Blastocystis* on the intestinal microbiota, these research studies give us an enormous advance in the knowledge of this microorganism and its potential behavior as a pathobiont, commensal, mutualist, or even a probiotic in the human intestine that could promotes regulation of the inflammatory immune response.

## Conclusion and personal perspectives

An undoubted fact in relation to *Blastocystis* is its interaction with the host at an immunological level and microbiota. It has been said a lot not only about its actual role in disease conditions (e.g., IBS and IBD) causing some gastrointestinal symptoms (e.g., diarrhea, vomiting, bloating, constipation, and abdominal pain) but also about merely being an organism that infects and establishes itself without causing any harm or even being beneficial to the host. The existence of many studies on *Blastocystis* trying to clarify and define the real character of *Blastocystis* as a pathogen, non-pathogen, commensal, mutualist, or even as an engineer of the host gut, has not been successful, so this controversy will continue and, hopefully, it will be clarified soon. For the moment, with the current information on *Blastocystis*, we can say that it interferes with or modifies the intestinal inflammatory immune response and the structure of the intestinal microbiota of the host. *Blastocystis* behavior in the host–parasite relationship appears to be a beneficial protist in the gut, shaping bacterial populations profiles associated to healthy intestinal microbiota rather than a pathogenic organism. Nevertheless, there are some recent pieces of evidence suggesting that *Blastocystis*, under particular circumstances, might display a pathogenic behavior.

There is a long way to go in the study of *Blastocystis*, and our personal interest about this microorganism is to establish how it acts in the modulation of the microbiota, because it may have a potential role as an intestinal probiotic. In this way, we could also determine its non-pathogenicity in vulnerable infected groups, whose immune status makes them susceptible to damage, and provide valuable information regarding the beneficial role of *Blastocystis* rather than just assuming the harmful behavior of this microorganism for humans.

## Author contributions

LR-V and CX conceptualized the review content. LR-V and CX wrote the first draft of the manuscript. TP-B participated in writing of specific paragraphs. LR-V, CX, PM, AS-V, EG, HP-J and TP-B edited the manuscript. EH, OP-R, MN-R, AP, and MZ participated in editing of specific paragraphs and re-reading of the text. All authors contributed to the article and approved the submitted version.

## Funding

The present work was supported by grant numbers IN217821 and IV200420 from PAPIIT- DGAPA, Universidad Nacional Autónoma de México (UNAM).

## Acknowledgments

The authors highly appreciate the informatics assistance of Angélica Serrano-Ahumada and Marco E. Gudiño-Zayas in the preparation of the manuscript.

## Conflict of interest

The authors declare that the research was conducted in the absence of any commercial or financial relationships that could be construed as a potential conflict of interest.

## Publisher’s note

All claims expressed in this article are solely those of the authors and do not necessarily represent those of their affiliated organizations, or those of the publisher, the editors and the reviewers. Any product that may be evaluated in this article, or claim that may be made by its manufacturer, is not guaranteed or endorsed by the publisher.

## References

[B1] Abdel-Hafeez E. H. Ahmad A. K. Abdelgelil N. H. Abdellatif M. Z. M. Kamal A. M. Hassanin K. M. A. . (2016). Immunopathological assessments of human blastocystis spp. in experimentally infected immunocompetent and immunosuppresed mice. Parasitol. Res. 115, 2061–2071. doi: 10.1007/s00436-016-4951-3 26860840

[B2] Abdelouhab K. Rafa H. Toumi R. Bouaziz S. Medjeber O. Touil-Boukoffa C. (2012). Mucosal intestinal alteration in experimental colitis correlates with nitric oxide production by peritoneal macrophages: Effect of probiotics and prebiotics. immunopharmacol. Immunotoxicol. 34, 590–597. doi: 10.3109/08923973.2011.641971 22211319

[B3] Alfellani M. A. Stensvold C. R. Vidal-Lapiedra A. Onuoha E. S. U. Fagbenro-Beyioku A. F. Clark C. G. (2013). Variable geographic distribution of blastocystis subtypes and its potential implications. Acta Trop. 126, 11–18. doi: 10.1016/j.actatropica.2012.12.011 23290980

[B4] Alzate J. F. Toro-Londoño M. Cabarcas F. Garcia-Montoya G. Galvan-Diaz A. (2020). Contrasting microbiota profiles observed in children carrying either blastocystis spp. or the commensal amoebas entamoeba coli or endolimax nana. Sci. Rep. 10, 15354. doi: 10.1038/s41598-020-72286-y 32948808 PMC7501860

[B5] Andersen L. O. Bonde I. Nielsen H. B. Stensvold C. R. (2015). A retrospective metagenomics approach to studying blastocystis. FEMS Microbiol. Ecol. 91, 1–9. doi: 10.1093/femsec/fiv072 26130823

[B6] Audebert C. Even G. Cian A. Loywick A. Merlin S. Viscogliosi E. (2016). Colonization with the enteric protozoa Blastocystis is associated with increased diversity of human gut bacterial microbiota. Sci. Rep. 6, 25255. doi: 10.1038/srep25255 27147260 PMC4857090

[B7] Aynur Z. E. Güçlü Ö. Yıldız İ. Aynur H. Ertabaklar H. Bozdoğan B. . (2019). Molecular characterization of blastocystis in cattle in Turkey. Parasitol. Res. 118, 1055–1059. doi: 10.1007/s00436-019-06243-8 30739165

[B8] Azizian M. Basati G. Abangah G. Mahmoudi M. R. Mirzaei A. (2016). Contribution of blastocystishominis subtypes and associated inflammatory factors in development of irritable bowel syndrome. Parasitol. Res. 115, 2003–2009. doi: 10.1007/s00436-016-4942-4 26841770

[B9] Bancroft A. J. Hayes K. S. Grencis R. K. (2012). Life on the edge: the balance between macrofauna, microflora and host immunity. Trends Parasitol. 28, 93–98. doi: 10.1016/j.pt.2011.12.001 22257556

[B10] Barash N. R. Maloney J. G. Singer S. M. Dawson S. C. (2017). Giardia alters commensal microbial diversity throughout the murine gut. Infect. Immun. 85, e00948–e00916. doi: 10.1128/IAI.00948-16 28396324 PMC5442636

[B11] Beghini F. Pasolli E. Truong T. D. Putignani L. Cacciò S. M. Segata N. (2017). Large-Scale comparative metagenomics of blastocystis, a common member of the human gut microbiome. ISME J. 11, 2848–2863. doi: 10.1038/ismej.2017.139 28837129 PMC5702742

[B12] Begue B. Verdier J. Rieux-Laucat F. Goulet O. Morali A. Canioni D. . (2011). Defective IL10 signaling defining a subgroup of patients with inflammatory bowel disease. Am. J. Gastroenterol. 106, 1544–1555. doi: 10.1038/ajg.2011.112 21519361

[B13] Belkaid Y. Hand T. W. (2014). Role of the microbiota in immunity and inflammation. Cell 157, 121–141. doi: 10.1016/j.cell.2014.03.011 24679531 PMC4056765

[B14] Billy V. Lhotská Z. Jirků M. Kadlecová O. Frgelecová L. Parfrey L. W. . (2021). Blastocystis colonization alters the gut microbiome and, in some cases, promotes faster recovery from induced colitis. Front. Microbiol. 12. doi: 10.3389/fmicb.2021.641483 PMC8058373 33897648

[B15] Brown E. M. Sadarangani M. Finlay B. B. (2013). The role of the immune system in governing host-microbe interactions in the intestine. Nat. Immunol. 14, 660–667. doi: 10.1038/ni.2611 23778793

[B16] Burgess S. L. Petri W. A. (2016). The intestinal bacterial microbiome and e. histolytica infection. Curr. Trop. Med. Rep. 3, 71–74. doi: 10.1007/s40475-016-0083-1 27525214 PMC4967426

[B17] Caballero S. Pamer E. G. (2015). Microbiota-mediated inflammation and antimicrobial defense in the intestine. Annu. Rev. Immunol. 33, 227–256. doi: 10.1146/annurev-immunol-032713-120238 25581310 PMC4540477

[B18] Candela M. Biagi E. Turroni S. Vitali B. Brigidi P. (2008). Mechanisms involved in the intestinal interaction between host and bifidobacteria. Microb. Ecol. Health Dis. 20, 189–192. doi: 10.1080/08910600802333483

[B19] Carasi P. Racedo S. M. Jacquot C. Romanin D. E. Serradell M. A. Urdaci M. C. (2015). Impact of kefir derived lactobacillus kefiri on the mucosal immune response and gut microbiota. J. Immunol. Res. 2015, 1–12. doi: 10.1155/2015/361604 PMC4355334 25811034

[B20] Castañeda S. Muñoz M. Villamizar X. Hernández P. C. Vásquez L. R. Tito R. Y. . (2020). Microbiota characterization in blastocystis-colonized and blastocystis-free school-age children from Colombia. Parasitol. Vectors 13, 521. doi: 10.1186/s13071-020-04392-9 PMC7565366 33066814

[B21] Cheng H. S. Guo Y. L. Shin J. W. (2003). Hematological effects of blastocystis hominis infection in male foreign workers in Taiwan. Parasitol. Res. 90, 48–51. doi: 10.1007/s00436-002-0804-3 12743803

[B22] Cho I. Blaser M. J. (2012). The human microbiome: at the interface of health and disease. Nat. Rev. Genet. 13, 260–270. doi: 10.1038/nrg3182 22411464 PMC3418802

[B23] Cinek O. Polackova K. Odeh R. Alassaf A. Kramná L. Ibekwe M. U. . (2021). Blastocystis in the faeces of children from six distant countries: prevalence, quantity, subtypes and the relation to the gut bacteriome. Parasitol. Vectors 14, 399. doi: 10.1186/s13071-021-04859-3 PMC8359624 34384477

[B24] Deng L. Tan K. S. W. (2022). Interactions between blastocystis subtype ST4 and gut microbiota *in vitro* . Parasitol. Vectors 15, 80. doi: 10.1186/s13071-022-05194-x PMC8902775 35260166

[B25] Deng L. Wojciech L. Gascoigne N. R. J. Peng G. Tan K. S. W. (2021). New insights into the interactions between blastocystis, the gut microbiota, and host immunity. PloS Pathog. 17, e1009253. doi: 10.1371/journal.ppat.1009253 33630979 PMC7906322

[B26] Deng L. Wojciech L. Png C. W. Koh E. Y. Aung T. T. Kioh D. Y. Q. . (2022). Experimental colonization with blastocystis ST4 is associated with protective immune responses and modulation of gut microbiome in a DSS-induced colitis mouse model. Cell. Mol. Life Sci. 79, 245. doi: 10.1007/s00018-022-04271-9 35435504 PMC9016058

[B27] Derrien M. van Passel M. W. J. van de Bovenkamp J. H. B. Schipper R. de Vos W. Dekker J. (2010). Mucin-bacterial interactions in the human oral cavity and digestive tract. Gut Microbes 1, 254–268. doi: 10.4161/gmic.1.4.12778 21327032 PMC3023607

[B28] Di Cristanziano V. Farowski F. Berrilli F. Santoro M. Di Cave D. Glé C. . (2021). Analysis of human gut microbiota composition associated to the presence of commensal and pathogen microorganisms in côte d’Ivoire. Microorganisms 9, 1763. doi: 10.3390/microorganisms9081763 34442844 PMC8400437

[B29] Domínguez-Márquez M. V. Guna R. Muñoz C. Gómez-Muñoz M. T. Borrás R. (2009). High prevalence of subtype 4 among isolates of blastocystis hominis from symptomatic patients of a health district of Valencia (Spain). Parasitol. Res. 105, 949–955. doi: 10.1007/s00436-009-1485-y 19471964

[B30] Eroglu F. Genc A. Elgun G. Koltas I. S. (2009). Identification of blastocystis hominis isolates from asymptomatic and symptomatic patients by PCR. Parasitol. Res. 105, 1589–1592. doi: 10.1007/s00436-009-1595-6 19685075

[B31] Even G. Lokmer A. Rodrigues J. Audebert C. Viscogliosi E. Ségurel L. . (2021). Changes in the human gut microbiota associated with colonization by blastocystis sp. and entamoeba spp. in non-industrialized populations. Front. Cell. Infect. Microbiol. 11. doi: 10.3389/fcimb.2021.533528 PMC8013780 33816323

[B32] Everard A. Belzer C. Geurts L. Ouwerkerk J. P. Druart C. Bindels L. B. . (2013). Cross-talk between akkermansia muciniphila and intestinal epithelium controls diet-induced obesity. Proc. Natl. Acad. Sci. 110, 9066–9071. doi: 10.1073/pnas.1219451110 23671105 PMC3670398

[B33] Fontanelli Sulekova L. Gabrielli S. Furzi F. Milardi G. L. Biliotti E. De Angelis M. . (2019). Molecular characterization of blastocystis subtypes in HIV-positive patients and evaluation of risk factors for colonization. BMC Infect. Dis. 19, 876. doi: 10.1186/s12879-019-4537-7 31640585 PMC6805496

[B34] Forsell J. Bengtsson-Palme J. Angelin M. Johansson A. Evengård B. Granlund M. (2017). The relation between blastocystis and the intestinal microbiota in Swedish travellers. BMC Microbiol. 17, 231. doi: 10.1186/s12866-017-1139-7 29228901 PMC5725903

[B35] Gabrielli S. Furzi F. Fontanelli Sulekova L. Taliani G. Mattiucci S. (2020). Occurrence of blastocystis-subtypes in patients from Italy revealed association of ST3 with a healthy gut microbiota. Parasite Epidemiol. Control 9, e00134. doi: 10.1016/j.parepi.2020.e00134 32258445 PMC7096745

[B36] Gause W. C. Rothlin C. Loke P. (2020). Heterogeneity in the initiation, development and function of type 2 immunity. Nat. Rev. Immunol. 20, 603–614. doi: 10.1038/s41577-020-0301-x 32367051 PMC9773851

[B37] Giacometti A. Cirioni O. Fiorentini A. Fortuna M. Scalise G. (1999). Irritable bowel syndrome in patients with blastocystis hominis infection. Eur. J. Clin. Microbiol. Infect. Dis. 18, 436–439. doi: 10.1007/s100960050314 10442423

[B38] Glendinning L. Nausch N. Free A. Taylor D. W. Mutapi F. (2014). The microbiota and helminths: sharing the same niche in the human host. Parasitology 141, 1255–1271. doi: 10.1017/S0031182014000699 24901211

[B39] Glocker E.-O. Kotlarz D. Boztug K. Gertz E. M. Schäffer A. A. Noyan F. . (2009). Inflammatory bowel disease and mutations affecting the interleukin-10 receptor. N. Engl. J. Med. 361, 2033–2045. doi: 10.1056/NEJMoa0907206 19890111 PMC2787406

[B40] Goodrich J. K. Davenport E. R. Beaumont M. Jackson M. A. Knight R. Ober C. . (2016). Genetic determinants of the gut microbiome in UK twins. Cell Host Microbe 19, 731–743. doi: 10.1016/j.chom.2016.04.017 27173935 PMC4915943

[B41] Gutzeit C. Magri G. Cerutti A. (2014). Intestinal IgA production and its role in host-microbe interaction. Immunol. Rev. 260, 76–85. doi: 10.1111/imr.12189 24942683 PMC4174397

[B42] Iebba V. Santangelo F. Totino V. Pantanella F. Monsia A. Di Cristanziano V. . (2016). Gut microbiota related to giardia duodenalis, entamoeba spp. and blastocystis hominis infections in humans from côte d’Ivoire. J. Infect. Dev. Ctries. 10, 1035–1041. doi: 10.3855/jidc.8179 27694739

[B43] Iebba V. Totino V. Gagliardi A. Santangelo F. Cacciotti F. Trancassini M. . (2016a). Eubiosis and dysbiosis: the two sides of the microbiota. New Microbiol. 39, 1–12. 26922981

[B44] Jiménez P. A. Jaimes J. E. Ramírez J. D. (2019). A summary of blastocystis subtypes in north and south America. Parasitol. Vectors 12, 376. doi: 10.1186/s13071-019-3641-2 PMC6664531 31358042

[B45] Johansen F.-E. Pekna M. Norderhaug I. N. Haneberg B. Hietala M. A. Krajci P. . (1999). Absence of epithelial immunoglobulin a transport, with increased mucosal leakiness, in polymeric immunoglobulin Receptor/Secretory component–deficient mice. J. Exp. Med. 190, 915–922. doi: 10.1084/jem.190.7.915 10510081 PMC2195652

[B46] Jonkers D. (2003). Probiotics and inflammatory bowel disease. J R Soc Med. 96, 5:167–171. PMC539443 12668702

[B47] Keragala C. B. Medcalf R. L. (2021). Plasminogen: an enigmatic zymogen. Blood 137, 2881–2889. doi: 10.1182/blood.2020008951 33735914

[B48] Kim M.-J. Lee Y. J. Kim T.-J. Won E. J. (2021). Gut microbiome profiles in colonizations with the enteric Protozoa blastocystis in Korean populations. Microorganisms 10, 34. doi: 10.3390/microorganisms10010034 35056483 PMC8777631

[B49] Kodio A. Coulibaly D. Koné A. K. Konaté S. Doumbo S. Guindo A. . (2019). Blastocystis colonization is associated with increased diversity and altered gut bacterial communities in healthy malian children. Microorganisms 7, 649. doi: 10.3390/microorganisms7120649 PMC6956266 31817168

[B50] Kurm V. van der Putten W. H. Weidner S. Geisen S. Snoek B. L. Bakx T. . (2019). Competition and predation as possible causes of bacterial rarity. Environ. Microbiol. 21, 1356–1368. doi: 10.1111/1462-2920.14569 30803145 PMC6850713

[B51] Lappan R. Classon C. Kumar S. Singh O. P. de Almeida R. V. Chakravarty J. . (2019). Meta-taxonomic analysis of prokaryotic and eukaryotic gut flora in stool samples from visceral leishmaniasis cases and endemic controls in bihar state India. PLoS Negl. Trop. Dis. 13, e0007444. doi: 10.1371/journal.pntd.0007444 31490933 PMC6750594

[B52] Lim M. X. Png C. W. Tay C. Y. B. Teo J. D. W. Jiao H. Lehming N. . (2014). Differential regulation of proinflammatory cytokine expression by mitogen-activated protein kinases in macrophages in response to intestinal parasite infection. Infect. Immun. 82, 4789–4801. doi: 10.1128/IAI.02279-14 25156742 PMC4249314

[B53] Ling X. Linglong P. Weixia D. Hong W. (2016). Protective effects of bifidobacterium on intestinal barrier function in LPS-induced enterocyte barrier injury of caco-2 monolayers and in a rat NEC model. PLoS One 11, e0161635. doi: 10.1371/journal.pone.0161635 27551722 PMC4995054

[B54] Long H. Handschack A. König W. Ambrosch A. (2001). Blastocystis hominis modulates immune responses and cytokine release in colonic epithelial cells. Parasitol. Res. 87, 1029–1030. doi: 10.1007/s004360100494 11763434

[B55] Maier T. V. Lucio M. Lee L. H. VerBerkmoes N. C. Brislawn C. J. Bernhardt J. . (2017). Impact of dietary resistant starch on the human gut microbiome, metaproteome, and metabolome. mBio 8, e01343–e01317. doi: 10.1128/mBio.01343-17 29042495 PMC5646248

[B56] Maloney J. G. Lombard J. E. Urie N. J. Shivley C. B. Santin M. (2019). Zoonotic and genetically diverse subtypes of blastocystis in US pre-weaned dairy heifer calves. Parasitol. Res. 118, 575–582. doi: 10.1007/s00436-018-6149-3 30483890

[B57] Matz C. Kjelleberg S. (2005). Off the hook – how bacteria survive protozoan grazing. Trends Microbiol. 13, 302–307. doi: 10.1016/j.tim.2005.05.009 15935676

[B58] McCoy K. D. Stoel M. Stettler R. Merky P. Fink K. Senn B. M. . (2008). Polyclonal and specific antibodies mediate protective immunity against enteric helminth infection. Cell Host Microbe 4, 362–373. doi: 10.1016/j.chom.2008.08.014 18854240

[B59] Mirza H. Teo J. D. W. Upcroft J. Tan K. S. W. (2011). A rapid, high-throughput viability assay for blastocystis spp. reveals metronidazole resistance and extensive subtype-dependent variations in drug susceptibilities. Antimicrob. Agents Chemother. 55, 637–648. doi: 10.1128/AAC.00900-10 21098237 PMC3028762

[B60] Moon C. Baldridge M. T. Wallace M. A. Burnham C.-A. D. Virgin H. W. Stappenbeck T. S. (2015). Vertically transmitted faecal IgA levels determine extra-chromosomal phenotypic variation. Nature 521, 90–93. doi: 10.1038/nature14139 25686606 PMC4425643

[B61] Moosavi A. Haghighi A. Mojarad E. N. Zayeri F. Alebouyeh M. Khazan H. . (2012). Genetic variability of blastocystis sp. isolated from symptomatic and asymptomatic individuals in Iran. Parasitol. Res. 111, 2311–2315. doi: 10.1007/s00436-012-3085-5 22948205

[B62] Muñoz Yañez C. M. Hernández A. M. Sandoval A. M. Domínguez M. A. M. Muñiz S. A. Z. Gómez J. O. G. (2021). Prevalence of blastocystis and its association with Firmicutes/Bacteroidetes ratio in clinically healthy and metabolically ill subjects. BMC Microbiol. 21, 339. doi: 10.1186/s12866-021-02402-z 34895145 PMC8665487

[B63] Nagel R. Traub R. J. Allcock R. J. N. Kwan M. M. S. Bielefeldt-Ohmann H. (2016). Comparison of faecal microbiota in blastocystis-positive and blastocystis-negative irritable bowel syndrome patients. Microbiome 4, 47. doi: 10.1186/s40168-016-0191-0 27580855 PMC5007835

[B64] Nieves-Ramírez M. E. Partida-Rodríguez O. Laforest-Lapointe I. Reynolds L. A. Brown E. M. Valdez-Salazar A. . (2018). Asymptomatic intestinal colonization with protist blastocystis is strongly associated with distinct microbiome ecological patterns. mSystems 3, e00007–e00018. doi: 10.1128/mSystems.00007-18 29963639 PMC6020473

[B65] Nishida A. Inoue R. Inatomi O. Bamba S. Naito Y. Andoh A. (2018). Gut microbiota in the pathogenesis of inflammatory bowel disease. Clin. J. Gastroenterol. 11, 1–10. doi: 10.1007/s12328-017-0813-5 29285689

[B66] Nourrisson C. Scanzi J. Brunet J. Delbac F. Dapoigny M. Poirier P. (2021). Prokaryotic and eukaryotic fecal microbiota in irritable bowel syndrome patients and healthy individuals colonized with blastocystis. Front. Microbiol. 12. doi: 10.3389/fmicb.2021.713347 PMC8486285 34603241

[B67] Nourrisson C. Scanzi J. Pereira B. NkoudMongo C. Wawrzyniak I. Cian A. . (2014). Blastocystis is associated with decrease of fecal microbiota protective bacteria: Comparative analysis between patients with irritable bowel syndrome and control subjects. PloS One 9, e111868. doi: 10.1371/journal.pone.0111868 25365580 PMC4218853

[B68] O’Brien Andersen L. Karim A. B. Roager H. M. Vigsnæs L. K. Krogfelt K. A. Licht T. R. . (2016). Associations between common intestinal parasites and bacteria in humans as revealed by qPCR. Eur. J. Clin. Microbiol. Infect. Dis. 35, 1427–1431. doi: 10.1007/s10096-016-2680-2 27230509

[B69] Ohkusa T. Yoshida T. Sato N. Watanabe S. Tajiri H. Okayasu I. (2009). Commensal bacteria can enter colonic epithelial cells and induce proinflammatory cytokine secretion: a possible pathogenic mechanism of ulcerative colitis. J. Med. Microbiol. 58, 535–545. doi: 10.1099/jmm.0.005801-0 19369513 PMC2887547

[B70] Ott S. J. (2004). Reduction in diversity of the colonic mucosa associated bacterial microflora in patients with active inflammatory bowel disease. Gut 53, 685–693. doi: 10.1136/gut.2003.025403 15082587 PMC1774050

[B71] Ott S. J. Plamondon S. Hart A. Begun A. Rehman A. Kamm M. A. . (2008). Dynamics of the mucosa-associated flora in ulcerative colitis patients during remission and clinical relapse. J. Clin. Microbiol. 46, 3510–3513. doi: 10.1128/JCM.01512-08 18701655 PMC2566070

[B72] Pandey P. K. Verma P. Marathe N. Shetty S. Bavdekar A. Patole M. S. . (2015). Prevalence and subtype analysis of blastocystis in healthy Indian individuals. Infect. Genet. Evol. 31, 296–299. doi: 10.1016/j.meegid.2015.02.012 25701123

[B73] Parfrey L. W. Walters W. A. Knight R. (2011). Microbial eukaryotes in the human microbiome: Ecology, evolution, and future directions. Front. Microbiol. 2. doi: 10.3389/fmicb.2011.00153 PMC3135866 21808637

[B74] Partida-Rodríguez O. Serrano-Vázquez A. Nieves-Ramírez M. E. Moran P. Rojas L. Portillo T. . (2017). Human intestinal microbiota: Interaction between parasites and the host immune response. Arch. Med. Res. 48, 690–700. doi: 10.1016/j.arcmed.2017.11.015 29290328

[B75] Pushpanathan P. Mathew G. S. Selvarajan S. Seshadri K. G. Srikanth P. (2019). Gut microbiota and its mysteries. Indian J. Med. Microbiol. 37, 268–277. doi: 10.4103/ijmm.IJMM_19_373 31745030

[B76] Puthia M. K. Vaithilingam A. Lu J. Tan K. S. W. (2005). Degradation of human secretory immunoglobulin a by blastocystis. Parasitol. Res. 97, 386–389. doi: 10.1007/s00436-005-1461-0 16151742

[B77] Reynolds L. A. Finlay B. B. Maizels R. M. (2015). Cohabitation in the intestine: Interactions among helminth parasites, bacterial microbiota, and host immunity. J. Immunol. 195, 4059–4066. doi: 10.4049/jimmunol.1501432 26477048 PMC4617609

[B78] Round J. L. Mazmanian S. K. (2010). Inducible Foxp3 + regulatory T-cell development by a commensal bacterium of the intestinal microbiota. Proc. Natl. Acad. Sci. 107, 12204–12209. doi: 10.1073/pnas.0909122107 20566854 PMC2901479

[B79] Rubtsov Y. P. Rasmussen J. P. Chi E. Y. Fontenot J. Castelli L. Ye X. . (2008). Regulatory T cell-derived interleukin-10 limits inflammation at environmental interfaces. Immunity 28, 546–558. doi: 10.1016/j.immuni.2008.02.017 18387831

[B80] Scanlan P. D. Stensvold C. R. Rajilić-Stojanović M. Heilig H. G. H. J. De Vos W. M. O’Toole P. W. . (2014). The microbial eukaryote blastocystis is a prevalent and diverse member of the healthy human gut microbiota. FEMS Microbiol. Ecol. 90, 326–330. doi: 10.1111/1574-6941.12396 25077936

[B81] Sekirov I. Russell S. L. Antunes L. C. M. Finlay B. B. (2010). Gut microbiota in health and disease. Physiol. Rev. 90, 859–904. doi: 10.1152/physrev.00045.2009 20664075

[B82] Shen D. Bai H. Li Z. Yu Y. Zhang H. Chen L. (2017). Positive effects of resistant starch supplementation on bowel function in healthy adults: a systematic review and meta-analysis of randomized controlled trials. Int. J. Food Sci. Nutr. 68, 149–157. doi: 10.1080/09637486.2016.1226275 27593182

[B83] Silberman J. D. Sogin M. L. Leipe D. D. Clark C. G. (1996). Human parasite finds taxonomic home. Nature 380, 398–398. doi: 10.1038/380398a0 8602239

[B84] Sjögren Y. M. Tomicic S. Lundberg A. Böttcher M. F. Björkstén B. Sverremark-Ekström E. . (2009). Influence of early gut microbiota on the maturation of childhood mucosal and systemic immune responses: Gut microbiota and immune responses. Clin. Exp. Allergy 39, 1842–1851. doi: 10.1111/j.1365-2222.2009.03326.x 19735274

[B85] Sobh M. Montroy J. Daham Z. Sibbald S. Lalu M. Stintzi A. . (2022). Tolerability and SCFA production after resistant starch supplementation in humans: a systematic review of randomized controlled studies. Am. J. Clin. Nutr. 115, 608–618. doi: 10.1093/ajcn/nqab402 34871343

[B86] Stensvold C. R. Alfellani M. A. Nørskov-Lauritsen S. Prip K. Victory E. L. Maddox C. . (2009). Subtype distribution of blastocystis isolates from synanthropic and zoo animals and identification of a new subtype. Int. J. Parasitol. 39, 473–479. doi: 10.1016/j.ijpara.2008.07.006 18755193

[B87] Stensvold C. R. Sørland B. A. Berg R. P. K. D. Andersen L. O. van der Giezen M. Bowtell J. L. . (2022). Stool microbiota diversity analysis of blastocystis-positive and blastocystis-negative individuals. Microorganisms 10, 326. doi: 10.3390/microorganisms10020326 35208781 PMC8878401

[B88] Stenzel D. J. Boreham P. F. (1996). Blastocystis hominis revisited. Clin. Microbiol. Rev. 9, 563–584. doi: 10.1128/CMR.9.4.563 8894352 PMC172910

[B89] Tan K. S. W. (2008). New insights on classification, identification, and clinical relevance of blastocystis spp. Clin. Microbiol. Rev. 21, 639–665. doi: 10.1128/CMR.00022-08 18854485 PMC2570156

[B90] Tan F. P. Y. Beltranena E. Zijlstra R. T. (2021). Resistant starch: Implications of dietary inclusion on gut health and growth in pigs: a review. J. Anim. Sci. Biotechnol. 12, 124. doi: 10.1186/s40104-021-00644-5 34784962 PMC8597317

[B91] The Human Microbiome Project Consortium (2012). Structure, function and diversity of the healthy human microbiome. Nature 486, 207–214. doi: 10.1038/nature11234 22699609 PMC3564958

[B92] Tito R. Y. Chaffron S. Caenepeel C. Lima-Mendez G. Wang J. Vieira-Silva S. . (2019). Population-level analysis of blastocystis subtype prevalence and variation in the human gut microbiota. Gut 68, 1180–1189. doi: 10.1136/gutjnl-2018-316106 30171064 PMC6582744

[B93] Toumi R. Abdelouhab K. Rafa H. Soufli I. Raissi-Kerboua D. Djeraba Z. . (2013). Beneficial role of the probiotic mixture ultrabiotique on maintaining the integrity of intestinal mucosal barrier in DSS-induced experimental colitis. Immunopharmacol. Immunotoxicol. 35, 403–409. doi: 10.3109/08923973.2013.790413 23638770

[B94] Vega L. Herrera G. Muñoz M. Patarroyo M. A. Maloney J. G. Santín M. . (2021). Gut microbiota profiles in diarrheic patients with co-occurrence of clostridioides difficile and blastocystis. PLoS One 16, e0248185. doi: 10.1371/journal.pone.0248185 33725006 PMC7963057

[B95] Vitetta L. Saltzman E. Nikov T. Ibrahim I. Hall S. (2016). Modulating the gut micro-environment in the treatment of intestinal parasites. J. Clin. Med. 5, 102. doi: 10.3390/jcm5110102 PMC5126799 27854317

[B96] Walsham N. Sherwood R. (2016). Fecal calprotectin in inflammatory bowel disease. Clin. Exp. Gastroenterol. 21, 21–29. doi: 10.2147/CEG.S51902 PMC4734737 26869808

[B97] Wu Z. Mirza H. Tan K. S. W. (2014). Intra-subtype variation in enteroadhesion accounts for differences in epithelial barrier disruption and is associated with metronidazole resistance in blastocystis subtype-7. PLoS Negl. Trop. Dis. 8, e2885. doi: 10.1371/journal.pntd.0002885 24851944 PMC4031124

[B98] Yason J. A. Ajjampur S. S. R. Tan K. S. W. (2016). Blastocystis isolate b exhibits multiple modes of resistance against antimicrobial peptide LL-37. Infect. Immun. 84, 2220–2232. doi: 10.1128/IAI.00339-16 27217421 PMC4962628

[B99] Yason J. A. Koh K. A. R. P. Tan K. S. W. (2018). Viability screen of LOPAC 1280 reveals phosphorylation inhibitor auranofin as a potent inhibitor of blastocystis subtype 1, 4, and 7 isolates. Antimicrob. Agents Chemother. 62, e00208–e00218. doi: 10.1128/AAC.00208-18 29866860 PMC6105778

[B100] Yason J. A. Liang Y. R. Png C. W. Zhang Y. Tan K. S. W. (2019). Interactions between a pathogenic blastocystis subtype and gut microbiota: *in vitro* and *in vivo* studies. Microbiome 7, 30. doi: 10.1186/s40168-019-0644-3 30853028 PMC6410515

[B101] Yatsunenko T. Rey F. E. Manary M. J. Trehan I. Dominguez-Bello M. G. Contreras M. . (2012). Human gut microbiome viewed across age and geography. Nature 486, 222–227. doi: 10.1038/nature11053 22699611 PMC3376388

[B102] Ze X. Duncan S. H. Louis P. Flint H. J. (2012). Ruminococcus bromii is a keystone species for the degradation of resistant starch in the human colon. ISME J. 6, 1535–1543. doi: 10.1038/ismej.2012.4 22343308 PMC3400402

